# Pregnancy and Childbirth: An Unexpected Cakewalk for a Mother With Beta Thalassemia Major Homozygous for IVS (G-C) Mutation

**DOI:** 10.7759/cureus.13872

**Published:** 2021-03-13

**Authors:** Mousumi D Ghosh, Mamta R Datta, Vinita Singh, Farah Rana

**Affiliations:** 1 Department of Obstetrics and Gynaecology, Tata Main Hospital, Jamshedpur, IND; 2 Department of Pathology, Tata Main Hospital, Jamshedpur, IND

**Keywords:** beta thalassemia major, ivs1-5(g-c) mutation, pregnancy

## Abstract

The thalassemias are the most common single-gene disorders of hemoglobin synthesis. The salient features of beta thalassemia major, in which both alleles of the HBB gene are affected, are transfusion dependency and iron overload. Although with advances in treatment, the life expectancy in such patients has increased, they have difficulty in conceiving. We report a case of pregnancy in a beta thalassemia major patient who was transfusion independent and had no iron overload. Genetic analysis revealed IVS 1-5 (G-C) mutation in the homozygous state which usually manifests in severe disease and blood transfusion dependency. On the contrary, she did not need blood transfusion, had a smooth antenatal period and a vaginal delivery at term with a favorable childbirth experience. This case report highlights complex genetic interplay and the role of fetal hemoglobin (HbF) enhancer loci which modulates HbF levels thereby raising total hemoglobin levels.

## Introduction

Thalassemia is one of the most common inherited genetic disorders. The key feature of beta thalassemia major is blood transfusion and iron overload [1]. Medical advances have led to increased life expectancy in these patients, with consequent increase in reproductive desire. Patients with beta thalassemia major rarely conceive due to hypogonadotropic hypogonadism [2]. With assisted conception, there are few case reports of pregnancy in beta thalassemia major. It is considered a very high-risk pregnancy for both mother and fetus. Among the various mutations, IVS 1-5 (G-C) is the severe form whose homozygosity results in severe disease. We report a case of homozygous IVS 1-5 (G-C) mutation who was not transfusion-dependent, conceived spontaneously, and had an uneventful maternal and fetal outcome.

## Case presentation

**Figure 1:**
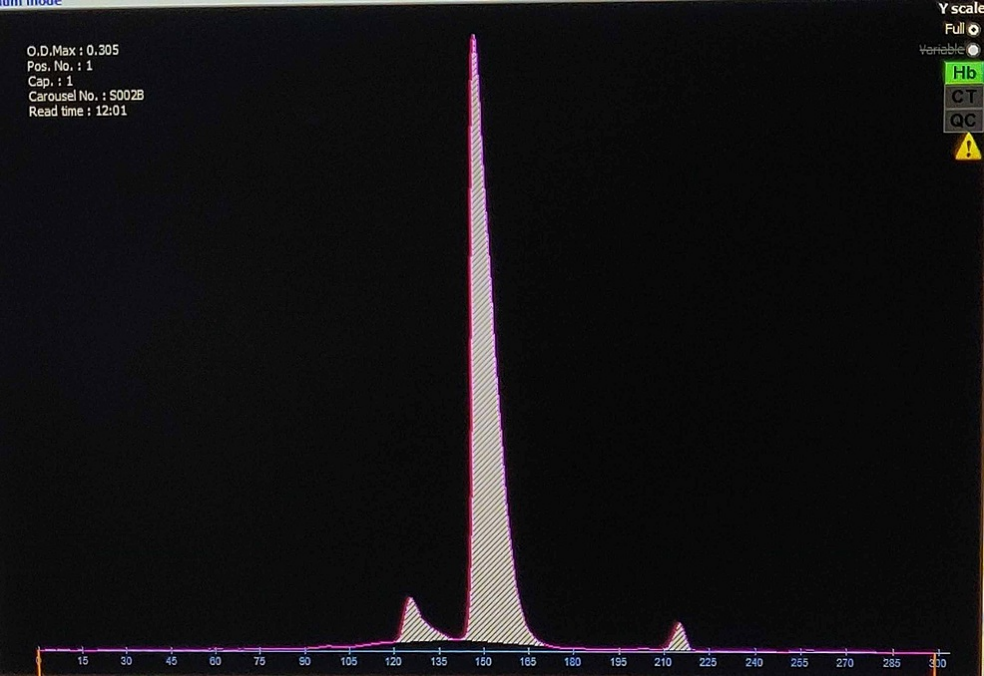
Capillary electrophoresis showing 91.7% HbF, 6.1% HbA, and 2.2% HbA2 levels, compatible with a diagnosis of thalassemia major

A 24-year-old primigravida attended an antenatal clinic at 14 weeks of pregnancy. She was a known case of beta thalassemia major, diagnosed two years back. Hemoglobin (Hb) electrophoresis showed HbA- 6.1%, HbF- 91.7% and HbA2- 2.2% (Figure 1).

Her height was 1.57 meters (m), weight 71 kilograms (kg) and body mass index was 28.8 kg/m². She had mild anemia with microcytic hypochromic blood picture. She had no history of blood transfusion and iron chelation therapy. This was a spontaneous conception with no history of infertility. The husband was tested for hemoglobinopathies and was found unaffected, hence the fetus was not subjected to prenatal testing. Her dating scan corresponded to the dates. Anomaly scan at 19 weeks of gestation showed a single live fetus with no anomalies.

Mutational analysis with amplification refractory mutation system polymerase chain reaction (ARMS PCR) using allele-specific primers to screen for beta thalassemia [IVS 1-5 (G-C), CD 41/42 (-CTTT), CD8/9 (+G), CD15 (G-A), CD30 (G-C)], CD6 (A-T) HbS and CD26 (G-A) HbE] was carried out to confirm the hemoglobinopathy. Results showed homozygous for beta thalassemia IVS 1-5 (G-C) mutation. She had regular antenatal check-ups in the outpatient dispensary and had mild anemia. The hematological parameters are described in Table 1.

**Table 1:** Hematological parameters at 16, 28 and 38 weeks of gestation ALT- alanine aminotransferase; AST- aspartate aminotransferase; ALP- alkaline phosphatase; PT INR- prothrombin time and international normalized ratio

	16 weeks	28 weeks	38 weeks
Hemoglobin	9.8 g/dl	9.6 g/dl	9.8 g/dl
Mean corpuscular volume (MCV)	68.2 fL		74.4 fL
Mean corpuscular hemoglobin (MCH)	21.3 pg		22.7 pg
Mean corpuscular hemoglobin concentration (MCHC)	31.3 pg		29.5 pg
Total leucocyte count	10,500 /uL		17000 /uL
Platelet count	156000 /uL		173000 /uL
Total bilirubin	6.12 mg/dl	4.61 mg/dl	2.56 mg/dl
Indirect bilirubin	5.38 mg/dl	3.72 mg/dl	1.37 mg/dl
ALT/AST/ALP	13/23.5/65.8 U/L	14.8/33.7/97.2 U/L	10/32/183 U/L
PT INR	1.05	1.08	1.06
Serum Iron	226 mcg/dL	322.4 mcg/dL	
Serum Ferritin	40 ng/mL	49.6 ng/mL	
Vitamin B12			114 ng/mL
Blood sugar	97 mg/dl	108 mg/dl	110 mg/dl
Thyroid stimulating hormone (TSH)	2.21 microIU/ml	3.1 microIU/ml	2.4 microIU/ml

Ultrasound abdomen showed multiple calculi in gall bladder with hepatosplenomegaly. Serial growth scans were done from 24 weeks of pregnancy which showed normal fetal growth. Her blood pressure and blood sugar levels were in the normal range throughout pregnancy. 

She was admitted to the labor room at 39 weeks 4 days of gestation with pain in abdomen and had spontaneous onset of labor. She delivered vaginally with an APGAR (Appearance, Pulse, Grimace, Activity, and Respiration) score of 9/10 and baby weight of 2.7 kg. It was an uneventful delivery with no maternal or fetal morbidity.

## Discussion

Patients with beta thalassemia major are transfusion-dependent and suffer from effects of iron overload. Of the various mutations, homozygosity for beta thalassemia IVS 1-5 (G-C) is the most common mutation in the Indian subcontinent and manifests in severe form. On the contrary, our patient had no history of blood transfusion before pregnancy and did not require one during pregnancy and labor.

These patients have reduced fertility and usually need assistance to conceive. After conception, there are increased risks to both mother and fetus, hence needing multidisciplinary treatment [3,4]. Maternal risks include cardiomyopathy, endocrinopathies (diabetes mellitus, hypothyroidism, hypoparathyroidism), severe maternal anemia, alloimmunization, viral infections, thrombosis and bone disturbances. These patients have iron overload and may need iron chelation in the second and third trimesters of pregnancy. Fetal risks include early pregnancy loss and growth restriction [5].

We report a case of thalassemia major who conceived spontaneously without any history of infertility. Our patient had mild anemia and hemolytic jaundice. The red cell indices mean corpuscular volume (MCV) and mean corpuscular hemoglobin (MCH) were below the cut-off of 80fL and 27pg with a high red cell distribution and normal red cell count. Her serum iron and ferritin levels were within range. There was no iron overload and she did not need any special monitoring. Ultrasound abdomen showed cholelithiasis for which no active intervention was needed. There were no maternal high-risk conditions and she had a smooth antenatal period. She had vaginal delivery at term with average birth weight. Mother and baby were discharged without any morbidity.

In this case, beta thalassemia major, homozygous for IVS 1-5 (G-C) mutation, showed features of thalassemia trait. A literature review showed a single case report by Bohara et al. [6] with similar presentation. Our case report is unique because the mutation is associated with pregnancy.

This extremely rare case report points to the fact that various modifiers can alter the severity of the mutation in the beta globin gene. HbF plays a major role in clinical presentation of beta thalassemia. Patients with high levels of HbF have less severe anemia [7]. Elevated levels of HbF modifies the balance between alpha and non-alpha globin chains and reduces the severity of beta thalassemia, both clinically and hematologically. The existence of HbF enhancer loci probably has changed the clinical picture in our case. Also various genetic interactions in the beta globin gene and outside the gene can affect the phenotypic presentation in beta thalassemia [8-11].

## Conclusions

The study of such cases emphasizes the fact that genetic interactions are complex and regulation of HbF has a role in clinical manifestation of beta thalassemia major. Simultaneous inheritance of some loci that modulate HbF levels causes high level of total hemoglobin, thereby requiring no blood transfusion. Understanding the regulation of globin gene expression and gene-gene interactions may open up new avenues for management of patients with beta thalassemia major.
